# Heterozygosity of the major histocompatibility complex predicts later self-reported pubertal maturation in men

**DOI:** 10.1038/s41598-021-99334-5

**Published:** 2021-10-06

**Authors:** Steven Arnocky, Carolyn Hodges-Simeon, Adam C. Davis, Riley Desmarais, Anna Greenshields, Robert Liwski, Ellen E. Quillen, Rodrigo Cardenas, S. Marc Breedlove, David Puts

**Affiliations:** 1 Nipissing University, North Bay, Canada; 2 Boston University, Boston, USA; 3 University of Ottawa, Ottawa, Canada; 4 Queen Elizabeth II Health Sciences Centre, Dalhousie University, Halifax, Canada; 5 Wake Forest School of Medicine, Winston-Salem, USA; 6 Pennsylvania State University, State College, USA; 7 Michigan State University, East Lansing, USA

**Keywords:** Evolutionary developmental biology, Evolutionary theory, Evolution

## Abstract

Individual variation in the age of pubertal onset is linked to physical and mental health, yet the factors underlying this variation are poorly understood. Life history theory predicts that individuals at higher risk of mortality due to extrinsic causes such as infectious disease should sexually mature and reproduce earlier, whereas those at lower risk can delay puberty and continue to invest resources in somatic growth. We examined relationships between a genetic predictor of infectious disease resistance, heterozygosity of the major histocompatibility complex (MHC), referred to as the human leukocyte antigen (HLA) gene in humans, and self-reported pubertal timing. In a combined sample of men from Canada (*n* = 137) and the United States (*n* = 43), MHC heterozygosity predicted later self-reported pubertal development. These findings suggest a genetic trade-off between immunocompetence and sexual maturation in human males.

## Introduction

Puberty in human males is a neuroendocrine process leading to sexual maturity characterized by a skeletal growth spurt, increased muscle mass, and the development of secondary sexual characteristics such as body and facial hair growth and deepening of the voice^1,2^. The age of pubertal onset^3,4^ shows substantial individual and population variation^5^ and is inversely associated with negative physical and mental health outcomes. Early puberty has been linked with increased cardiometabolic disease risk factors in adulthood such as higher body mass index^6^, fasting insulin, diastolic blood pressure, and decreased HDL, as well as elevated LDL, serum cholesterol, and triglyceride levels in men^7^. Furthermore, precocious puberty has been linked to prostate cancer risk^8,9^, less sleep^10^, angina and hypertension^11^, as well as an array of mental health disorders^10^. Yet, the factors that link variation in pubertal timing with health remain poorly understood.

Life history theory (LHT) is a theoretical framework aimed at explaining variation in sexual maturation as a result of trade-offs between the competing demands of growth, reproduction, and maintenance^12–15^. Trade-offs are especially acute during puberty because of competing investments in reproductive development and somatic growth^5,16^. For example, earlier pubertal development has been associated with both lower adult stature^5,17,18^, and with greater sex drive and interest in casual sex^19,20^, as well as higher reported number of lifetime sex partners^19^—associations that are consistent with a trade-off between investing in reproduction or continued somatic growth.

A core prediction of LHT is that the relative costs and benefits of reproductive maturation depend on the degree of extrinsic mortality risk; that is, when extrinsic mortality risk is high, earlier sexual maturation may offer greater reproductive benefits than continued growth^5,12,15,21–23^. Some circumstantial evidence supports this link. For instance, adopted children often suffer from infectious health conditions, and tend to develop earlier than children in either their host or originating country^24^. A key driver of extrinsic mortality risk is pathogen species richness in the environment^25,26^, which selects for heterozygosity on the major histocompatibility complex (MHC)^27^. MHC, referred to as the human leukocyte antigen (HLA) complex in humans, comprises a series of highly linked genes located on the short arm of chromosome 6. The classical HLA genes in this complex (HLA-A, HLA-B, HLA-C, HLA-DR, HLA-DQB1, HLA-DQA1, HLA-DPB1, HLA-DPA1) encode cell surface receptors that play a central role in immunological recognition of the self from the non-self (i.e., pathogens). Of the classical HLA genes, HLA-A, HLA-B, HLA-C (class I) and HLA-DRB1 (class II) are the most polymorphic loci according to the IPD-IMGT/HLA database^28,29^. As HLA gene expression is co-dominant, both maternally and paternally inherited alleles are expressed^30^. According to the heterozygote advantage hypothesis, MHC heterozygosity confers enhanced resistance to pathogens due to the ability to recognize a greater number of pathogenic antigens^27^.

Indeed, research has linked MHC heterozygosity with resistance to pathogens in both non-humans^31,32^ and humans^33,34^. Compared with homozygosity, heterozygosity is associated with slower progression of immunodeficiency virus-1 (HIV-1)^35^, lower likelihood of hepatitis C infection among liver transplant recipients^33^, and lower provirus load among individuals infected with the inflammatory disease human T cell lymphotropic virus type 1^34^. Specific MHC alleles have also been linked to resistance and susceptibility to infectious diseases^36–38^.

Direct evidence supporting the relationship between the MHC and pubertal development is limited. In one notable example, a recent epidemic of an infectious and highly fatal facial tumor disease among eastern Tasmanian devils was directly linked to diversity loss among MHC genes^39^. After 6 years of high adult mortality, this population showed a 16-fold higher chance of reaching sexual maturity at an earlier age than usual^26^—suggesting a pathway through which MHC homozygosity may be related to earlier puberty. Among humans, no studies have yet examined associations between MHC and pubertal timing, although recent evidence has linked women’s MHC homozygosity to earlier sexual debut^40^, which is itself related to earlier puberty^41^. However, this research did not examine pubertal development directly. A review of studies on infection and pubertal development showed that infection was associated with later breast development in human females, whereas evidence was inconsistent for development of genitalia and pubic hair^42^.

Although unreliable resource availability would seem to represent another extrinsic mortality risk factor favoring earlier sexual maturation and reproduction, these life history events are sufficiently constrained by energy availability that the reverse appears to be generally true: increased energy available for growth decreases the marginal costs of reproduction, and as survival improves in a population, pubertal development would be expected to accelerate^43^. Both energetics theory and stress suppression theory posit that energetic scarcity will predict a delay in reproduction^1^, and it is well established that malnutrition can delay pubertal development^44^. Some researchers have speculated that mortality risk might only affect pubertal timing when nutrition needs are met^45,46^. This dichotomy is well exemplified by research from 22 small scale societies showing that groups with more favorable environmental conditions exhibited earlier puberty, yet those with greater extrinsic mortality risk exhibited evidence of faster development and earlier reproduction^5^. Previous research suggested that energy-rich environments, such as those comprising modern Western industrialized nations, reduce the environmental effects and increase the genetic influence on individual differences in pubertal timing^47^. Accordingly, the goal in the present study was to examine MHC heterozygosity as a potential genetic correlate of later reported relative (compared to others the same age) and absolute pubertal timing in a combined sample of well-nourished Canadian and American men. In line with the LHT literature on pubertal timing, we tested the following hypothesis: Men who are more MHC heterozygous will report later relative pubertal development than those who are less heterozygous.

## Results

**Figure 1:**
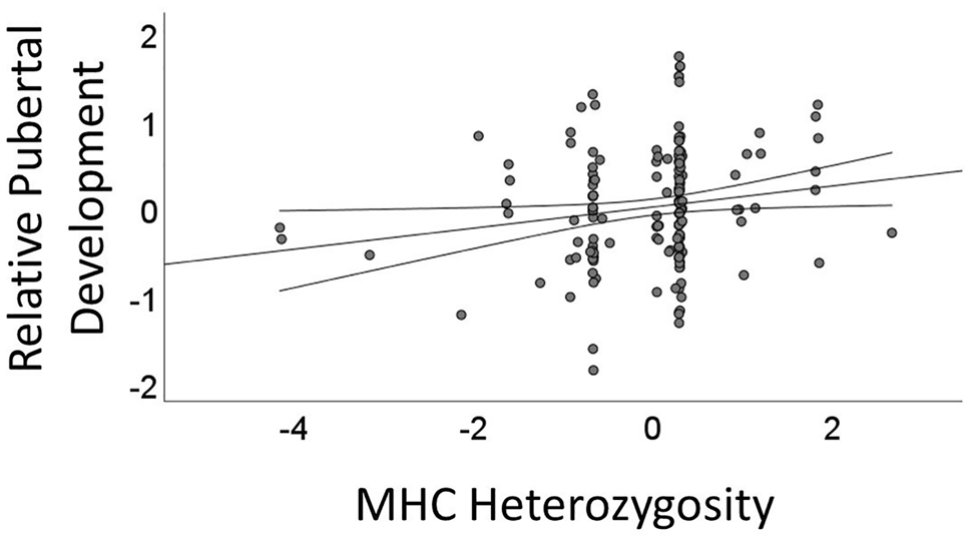
Partial regression plot. Best-fit line from least-square regression and 95% confidence interval for the relationship between z-transformed MHC heterozygosity residual values and self-reported relative pubertal development residual values in young men.

Two samples containing measures of MHC heterozygosity and relative and absolute pubertal development were combined to maximize statistical power. Because measures of MHC heterozygosity varied across studies, they were standardized using a z-transformation prior to combining data sets, resulting in a sample of 180 young adult men (Sample 1: 137 Canadians and Sample 2: 43 Americans). Higher scores on the pubertal development measures indicated later development, and a higher score on MHC heterozygosity reflected more heterozygosity. Participants mean reported relative pubertal development was in line with the scale midpoint (*M* = 3.06, *SD* = 0.64, Min = 1.00, Max = 4.80), and their absolute development occurred on average near age 14 years old (*M* = 13.70, *SD* = 1.85, Min = 1.58, Max = 20.00). First, we examined partial correlations among variables. Controlling for sample origin, age, and ethnicity (Caucasian versus other), results revealed that MHC heterozygosity correlated with relative pubertal development, *r*(*df* = 175) = 0.22, *p* = 0.003, such that men who were more MHC heterozygous reported pubertal development that was later than men who were more MHC homozygous. Conversely, MHC heterozygosity was unrelated to reports of absolute pubertal development, *r*(*df* = 175) = 0.10, *p* = 0.17. Results did not meaningfully change with the removal of control variables; MHC heterozygosity remained a correlate of relative, *r*(180) = 0.22, *p* = 0.003, but not absolute *r*(180) = 0.11, *p* = 0.16, pubertal development. Second, we conducted a multiple regression with MHC heterozygosity, sample source, participant age, and ethnicity as independent variables in step 1, MHC x sample source in step 2, and relative pubertal development as the dependent variable (see Table 1). Only MHC heterozygosity predicted relative pubertal development (see Fig. 1). Winsorizing outliers that were lower than ± 2SD from the mean on relative or absolute pubertal development did not meaningfully influence the results reported herein.

**Table 1:** Results of a multiple linear regression depicting the predictive relationship between MHC heterozygosity and relative pubertal development.

	Model 1				Model 2			
	*b*	*SE*	*t*	*p*	*b*	*SE*	*t*	*p*
MHC Heterozygosity	0.14	0.05	2.96	0.003	0.12	0.06	2.18	0.03
Sample	0.03	0.09	0.30	0.78	0.03	0.09	0.34	0.74
Ethnicity	0.11	0.17	0.66	0.51	0.12	0.17	0.73	0.47
Age	− 0.01	0.01	− 0.27	0.79	− 0.02	0.01	− 0.27	0.79
MHC Heterozygosity x Sample	–	–	–	–	0.04	0.06	0.67	0.50

*b* = unstandardized regression coefficient, *SE* = standard error, *t* = *t*-score, *p* = *p*-value.

## Discussion

Our results support the prediction that greater MHC heterozygosity, a genetic contributor to pathogen resistance^33,34^, predicts later pubertal timing. In a combined data set derived from two independent samples, MHC heterozygosity predicted relative, but not absolute, recalled puberty. Because males lack a salient, singular pubertal event like menarche, when considering retrospective reports relative pubertal timing may be more accurate because men may be better able to recall whether they matured earlier or later than their peers rather than the precise ages of pubertal events^48,49^.

Correlations between immunocompetence and pubertal timing could reflect the linked heritability of both traits, common developmental underpinnings^50^, or pleiotropic effects of MHC genes, which could influence both immunocompetence and sexual maturation. Indeed, some research has shown that MHC class II expression occurs alongside maturation of the adrenal cortex^51^. Interestingly, dehydroepiandrosterone (DHEA), which is produced by the adrenal cortex and affects aspects of reproductive development, has been implicated in immune function in humans and other species^52,53^.

LHT offers a framework to explain why immunocompetence and pubertal timing may be related at a functional level: Individuals with reduced extrinsic mortality risk due to lower vulnerability to pathogens may be able to continue growth and delay sexual maturation and reproduction. If so, then selection should favor mechanisms, potentially including pleiotropy and genetic linkage, that couple immunocompetence and the timing of sexual maturation. This possibility aligns with some research on intra-species differences in LH. For example, Tasmanian devil populations affected by an infectious facial tumor disease had a 16-fold higher chance of reaching sexual maturity at an earlier age than usual^26^. In a study of 22 small-scale human societies, populations with higher extrinsic mortality risk displayed earlier puberty and reproduction—as well as shorter adult height and life expectancy^5^.

Future work must reconcile research showing opposing patterns, such as among perinatal HIV infection and slower pubertal maturation^54^. Perhaps the distinction lies in genetic versus acquired factors affecting immunocompetence, or environmental factors (e.g., food energy availability of safety/survival rates), which might also influence luteinizing hormone (LH) release in diverse human populations^55^. For example, malnutrition has been linked to delayed pubertal maturation in humans^44^. Accordingly, future research should consider the role of energy availability in the environment as a potentially important moderator of the potential link between MHC and pubertal development. For instance, perhaps the influence of infectious burden on energy availability may be lower in populations with energy abundance and substantial health infrastructure, such as in Western industrialized nations.

Our findings also help explain why MHC heterozygous men have been found to be taller in adulthood^56^. Height is driven by long bone growth via chondrogenesis at the growth plate^57^, and epiphyseal fusion at puberty terminates growth. Later pubertal maturation allows more long bone growth before epiphyseal fusion, resulting in taller adult height^18^; therefore, heterozygous individuals may be taller because they begin puberty later. Future research could test whether pubertal timing mediates the relationship between MHC heterozygosity and adult height. It may be useful to examine the potential moderating role of early stressors in the environment to the MHC-pubertal timing link. From this perspective, developmental plasticity gives rise to an array of phenotypes that emerge in response to specific local social and ecological conditions^58^. These genetic variants are putatively adaptive insofar as they contribute to greater fitness in the environments in which they manifest. Accordingly, an interaction between HLA homozygosity and early life stressors may be a stronger predictor of pubertal timing than either variable alone.

Within the context of LHT, some researchers have predicted that greater investment in immunocompetence should correspond with later sexual maturation. Although previous research linking early pubertal maturation to a diverse range of health problems supports this notion, this is the first research to demonstrate a correlation between MHC heterozygosity and later recalled pubertal development. Such a link has important implications for understanding the development of puberty-linked physical and mental health outcomes. These results suggest that variation in genetic influences on pubertal timing may reflect a trade-off between somatic growth and maintenance and reproduction, at least in energy-rich environments. However, within the broader context of well-established positive links between environmental condition and earlier (rather than later) pubertal timing, these findings imply that understanding variability in reproductive effort will likely rely upon examining more complex interactions between genetics and local ecological condition.

## Method

### Sample 1 (Canada)

#### Participants

Undergraduate men (*N* = 137) between the ages of 18–39 (*M*_*age*_ = 22.71, *SD* = 4.70) were recruited through the university-wide research participation system. The sample was largely Caucasian (90%). As part of a larger study on men’s health and morphology, participants provided a blood sample and completed self-report questionnaire about their pubertal development. The protocol was approved by the Nipissing University Research Ethics Board (protocol # 100770) in accordance with the Canadian Tri-Council Policy Statement: Ethical Conduct for Research Involving Humans – TCPS 2. All participants provided written informed consent.

#### MHC heterozygosity

Genomic DNA was extracted from whole blood obtained by venipuncture and captured in EDTA tubes. HLA typing for loci HLA-A, HLA-B, and HLA-DRB1 was performed by the reverse sequence-specific oligonucleotide probe (RSSO) method using LABType™ SSO DNA typing kits (One Lambda, CA), as per the manufacturer’s instructions. HLA typing results were reported for HLA-A, HLA-B, and HLA-DRB1 loci, with heterozygosity first coded dichotomously (yes/no) for each locus and then calculated as a sum ranging between 0 and 3. In this sample, every participant was heterozygous on at least one locus, with 2% being heterozygous at one, 24% at two, and 74% at three loci, which is consistent with larger samples examining these markers^59^.

#### Pubertal development

In both samples, participants completed the Pubertal Development Scale^60^. Following previous research^49^ the measure was modified so that it could be answered retrospectively. The items cover timing of voice change, growth spurt, body hair growth, skin changes, spontaneous erections, nocturnal emissions, and development in general. The items are reported using relative and absolute scales. An example item from the relative development subscale is “Do you think you began growing facial hair and shaving any earlier or later than most other boys?” with response options ranging along a five-point scale from 1 = “much earlier” to 5 = “much later,” and a sixth option of “don’t know”. The corresponding item from the absolute subscale was “How old were you when you began growing facial hair? Please enter your response to the nearest month (Example: __ years and __ months)” with age being rounded up or down to the nearest year. Mean scores for each subscale were created, omitting responses where the participant was unsure. Both the relative (α = 0.77) and absolute (α = 0.83) pubertal development measures demonstrated good internal consistency, using Cronbach’s alpha. Some researchers have utilized the PDS by computing the relative subscale excluding two items: One which measures relative spontaneous erections, and one which measures nocturnal emissions, with the underlying rationale that this may be difficult to gauge relative to same-sex others^61^. Although we elected to retain use of the measure as it was designed, rerunning our analyses post-hoc excluding these items did not meaningfully alter the results reported herein. Given that internal consistencies did not meaningfully change with these items excluded, we elected to retain the items following the original measurement structure.

### Sample 2 (USA)

#### Participants

Undergraduate men (*N* = 50) between the ages of 18–25 (*M*_*age*_ = 19.30, *SD* = 1.54) with European-American Ancestry provided a DNA sample and completed the same measure of pubertal development as described in Sample 1. Again, both the relative (α = 0.74) and absolute (α = 0.68) pubertal development measures demonstrated acceptable internal consistency. Six men were missing data on their pubertal development (> 70%), and one did not provide a DNA sample, and were thus excluded from analyses (final *N* = 43; range = 18–25; *M*_*age*_ = 19.34, *SD* = 1.61). The protocol was approved by the Michigan State University Human Research Protection Programs Institutional Review Board (IRB#: 06–556). All participants provided written informed consent.

#### MHC Heterozygosity

DNA was extracted from blood from a finger prick stored on Whatman cards. The overall heterozygosity of the region was estimated using 10 short tandem repeat markers (STRs) in and near HLA genes located at 6p21.3. The HLA markers from the dbMHC database^62^ as well as previous published papers [63.64] were selected that flank the genes HLA-A, HLA-B, HLA-C, HLA-DRB1, HLA-DQA1, and HLA-DQB1. Markers were selected to be as close to the gene as possible without being in linkage disequilibrium with one another. Estimates of LD were based on the haplotype blocks identified in the literature^65,66^. Preference was given to the STRs with the highest expected frequency of heterozygotes in European Americans. The markers D6S2931, D6S2863, D6S2883, D6S2810, D6S2811, D6S2876, D6S510, D6S1666, D6S2664, and D6S2447 were selected.

To control for the overall level of heterozygosity in the genome, the subjects were also genotyped for the standard panel of CODIS markers^67^. The CODIS markers are a widely used panel for forensic investigations so the expected heterozygosities are relatively high and well established. Since these STRs are known to be neutral, they should represent a good estimate of each individual’s genome-wide heterozygosity. Individuals with call rates of less than 90% for either of the marker panels or individuals who are siblings of other subjects were previously removed from the sample. For each individual, the heterozygosity was calculated for both the MHC and the CODIS panels using genhet package in R to calculate heterozygosity using the Homozygosity by Locus (HL) method developed by Aparicio et al.^68,69^. In all analyses, the individual’s percent heterozygosity for the HLA markers is conditioned on the percent heterozygosity for the CODIS markers so that the effect of heterozygosity at other loci does not confound the relationship. Thus, positive MHC heterozygosity represents a more heterozygous MHC region than expected based on heterozygosity in the rest of an individual’s genome and a negative heterozygosity represents greater homozygosity in the MHC region than expected.

## Data Availability

Data from this research are available on the Open Science Framework (OSF) at https://osf.io/yckt6/.
